# Limitations of poster presentations reporting educational innovations at a major international medical education conference

**DOI:** 10.3402/meo.v18i0.20498

**Published:** 2013-02-19

**Authors:** Morris Gordon, Daniel Darbyshire, Aamir Saifuddin, Kavitha Vimalesvaran

**Affiliations:** 1 Faculty of Health and Social Care, University of Salford, Salford, UK; 2 Department of Paediatrics, Blackpool Victoria Hospital, Blackpool, UK; 3 Department of Urology, Salford Royal Hospital, Salford, UK; 4 Department of General Medicine, King's College Hospital, London, UK; 5 Department of General Surgery, King's College Hospital, London, UK

**Keywords:** patient safety, non-technical skills, human factors, adverse events

## Abstract

**Background:**

In most areas of medical research, the label of ‘quality’ is associated with well-accepted standards. Whilst its interpretation in the field of medical education is contentious, there is agreement on the key elements required when reporting novel teaching strategies. We set out to assess if these features had been fulfilled by poster presentations at a major international medical education conference.

**Methods:**

Such posters were analysed in four key areas: reporting of theoretical underpinning, explanation of instructional design methods, descriptions of the resources needed for introduction, and the offering of materials to support dissemination.

**Results:**

Three hundred and twelve posters were reviewed with 170 suitable for analysis. Forty-one percent described their methods of instruction or innovation design. Thirty-three percent gave details of equipment, and 29% of studies described resources that may be required for delivering such an intervention. Further resources to support dissemination of their innovation were offered by 36%. Twenty-three percent described the theoretical underpinning or conceptual frameworks upon which their work was based.

**Conclusions:**

These findings suggest that posters presenting educational innovation are currently limited in what they offer to educators. Presenters should seek to enhance their reporting of these crucial aspects by employing existing published guidance, and organising committees may wish to consider explicitly requesting such information at the time of initial submission.

## Background

Quality is a key concern in all fields of medical research. Within clinical medicine, there is a very clear hierarchy of research methods, with higher level methods likely to contribute more to the wider ‘clinical truth’. Through work by international organisations that promote systematic review methods, such as the Cochrane Collaboration, evidence can be consistently synthesised to support evidence-based medicine and enhance patient care.

In the world of medical education, the situation is far more complex and challenging. An article in the *British Medical Journal* several years ago sparked an active debate regarding the nature of quality within medical research (1). The authors concluded that research lacks methodological rigour. This led to responses from scholars in the field within the pages of this journal (2) who were concerned that medical education research ‘cannot be viewed in such a uni-dimensional way’, and that evidence should not be viewed in hierarchies of quality but should be selected like colours in a rich tapestry. Eva (3) describes this as ‘an endless oscillation between promoting the evolving empirically grounded approach and the associated criticisms of the accumulated findings’, concluding that quality in medical education research should be based on our understanding of the problems, rather than on whether or not a particular research methodology has been adopted. Questions such as ‘how’, ‘why’, and ‘when’ education is effective are increasingly being sought from researchers (4).

The ‘Best Evidence Medical Education’ (BEME) collaboration has endeavoured to address such issues and has produced materials for data extraction that seek to view quality in a multi-dimensional manner. Other than supporting evidence synthesis, such materials support the view that quality should not be based on a single arbiter and offer insights into gold standard elements of reporting educational innovations.

Whilst such ideas surrounding the reporting of medical education research are clearly widespread in the literature, it is unclear how much this debate is influencing those who are reporting on research. We set out to assess the quality of poster presentations describing educational innovations at a major international medical education conference from a multi-dimensional perspective.

## Methods

At the 2012 Association of Medical Education in Europe (AMEE) International Conference 2012 in Lyon, there were 636 poster presentations in English (5). A random sample of 50% of these was selected for inclusion in the assessment process. Presentations reporting a new innovation or method were included and data extracted using a *pro forma* (Appendix 1). The studies included were analysed in four key areas: reporting of theoretical underpinning, reporting of instructional design methods, describing of resources needed for introduction, and, finally, the offering of materials to support dissemination.

Each of the first 15 posters was assessed by two authors to assess concordance, which was 75%. The discrepancies were analysed, and assessment of a further 10 posters gave 88% concordance in the major assessment items. The remaining posters were evaluated by one author each. Any concerns regarding decisions were discussed between the authors and a consensus was reached.

## Results

A total of 312 posters (49%) were assessed. One-hundred and forty-two posters were excluded as they did not report a new educational innovation. This included 7 audits, 72 cross-sectional surveys, 14 narratives, 5 opinion pieces, and 44 service evaluations.

One hundred and seventy poster presentations were included within the analysis. Seventy of these (41%) described their methods of design, 56 (33%) gave details of equipment, and 49 (29%) described resources required. Sixty-one (36%) offered further resources to support dissemination of their innovation. Thirty-nine studies discussed theory or conceptual framework underpinning their work. The remaining 141 (77%) made little, if any, allusion to any such elements; they did often mention relevant literature, which may have implied an orientation to an appropriate framework, but this was not explicitly stated.

## Discussions

Poster presentations at international medical educational conferences enable the dissemination of descriptions of exciting innovations, even if the work has not become the focus of a full-scale research project. In this small study, it has been found that such reports are often lacking in key areas that may be associated with ‘quality’ in the context of educational research.

Before discussing these findings further, it is important to make clear that the authors recognise that the very element we have sought to assess is, by its very nature, not as simple as three or four criteria, as discussed above. In addition, such judgments are also subjective, with the perspectives of the reader often influencing the perceived quality of the research. Nevertheless, it is difficult to overlook that over half of the posters describing innovations offered no details regarding the resources or methods of design. Many focused on whether their intervention was effective, offering data regarding acceptability, changes in attitudes, or changes in knowledge or skills. However, given the relatively small scale or early stage of most of the reported developments, the authors believe that details facilitating dissemination of these, often impressive, innovations should be prioritised over the description of low-powered quantitative outcomes.

The lack of details regarding the theoretical orientation or the consideration of appropriate conceptual frameworks was the starkest finding. These play an essential role in identifying the nature of educational problems and in formulating solutions or designing studies. They help clarify and magnify the issues at hand. The use of frameworks allows authors to be mindful of the assumptions and foundations of their work and makes the process transparent for the reader. For those without an educational background, this may be a new concept, but it is key for informing those reviewing such work and to support future research. In many cases, it is likely that the details are available but simply not presented.

Whilst considering these findings, it is must be noted that this is a small study based on a single conference, and only a sample of posters were reviewed. Also, whilst checks were made for concordance, not all presentations were reviewed by two authors. In addition, the authors have focussed on studies reporting educational interventions, but clearly there are many other worthwhile forms of research and innovation that have not been considered within this definition. Finally, our definitions and judgements are ultimately subjective, though given the magnitude of our respective findings, they most likely provide an appropriate approximation.

## Conclusions

These findings suggest that posters presenting educational innovation are currently limited in what they offer for educators. Presenters should seek to enhance their reporting to include these important elements. In addition, conference organising committees may wish to consider explicitly requesting such information at the time of initial submission to support the useful dissemination of these works to their attendees.

## Appendix 1: Assessment of poster presentations – AMEE 2012

**Which session was this poster from (highlight in bold)**

2W Posters: Career Choice

2X Posters: The Education Environment

2Y Posters: Continuing Professional Development

2Z Posters: Outcome Based Education

2AA Posters: Clinical Skills

2BB Posters: Written Assessment

3W Posters: Simulation

3X Posters: Research and Evidence Based Medicine

3Y Posters: Postgraduate Training 1

3Z Posters: Problem Based Learning

3AA Posters: The Student in Difficulty

3BB Posters: Clinical Assessment

4W Posters: Faculty Development

4X Posters: Selection

4Y Posters: Postgraduate Training 2

4Z Posters: Curriculum Development

4AA Posters: Clinical Teaching 1

6V Meeting: AMEE Simulation Committee

6W Posters: The Teacher and Evaluation of the Teacher

6X Posters: Basic Sciences

6Y Posters: The Doctor as Teacher/Training the Surgeon

6Z Posters: Curriculum Development 2

6AA Posters: Clinical Teaching 2

6BB Posters: Feedback and Online Assessment

7W Posters: The Student as Teacher

7X Posters: Professionalism

7Y Posters: GP Education, Mentoring and Postgraduate Education

7Z Posters: Curriculum Evaluation

7AA Posters: Communication Skills

7BB Posters: Teaching and Learning Methods and Students’ Learning Styles

8W Posters: eLearning Case Studies 1

8X Posters: Interprofessional Education

8Y Posters: Health Promotion and Public Health

8Z Posters: Community Oriented Medical Education

8AA Posters: Lectures and Learning Resources

8BB Posters: Student Engagement and the Student as Teacher

9W Posters: eLearning Case Studies 2

9X Posters: Leadership/Management

9Y Posters: Reflection, Clinical Reasoning and Critical Thinking

9Z Posters: Team Based Learning/Case Based Learning

9AA Posters: Selection and The Student and Resident in Difficulty

9BB Posters: Students

10W Posters: Patient Safety

10X Posters: Ethics and Empathy

10Y Posters: Work Based Assessment

10Z Posters: Curriculum Evaluation and Electives

10AA Posters: Active and Student Centred Learning

10BB Posters: Assessment

**Was the poster reviewed or just abstract review?** (delete as appropriate) Poster Abstract

**What type of work does this describe?** (highlight in bold as appropriate)

RCT / Before and after trial / Action based / case control / Cohort / **- PLEASE CONTINUE**

Opinion / Audit / Service evaluation / descriptive / Narrative **- NO FURTHER INFORMATION REQUIRED**

**Is relevant educational theory or general theoretical underpinning discussed with this presentation?** YES – (please give details) / No

_________________________________________________________________________

_________________________________________________________________________

_________________________________________________________________________

**Are resources, design methods and equipment needed described?** YES (give details) / No / N/A

_________________________________________________________________________

_________________________________________________________________________

_________________________________________________________________________

**If yes, please state on a scale of 1–5 how useful this is, 1 being limited and not of great benefit, 5 supporting widespread replication**.

_________________________________

**If the presentation could potentially be disseminated, are materials given to support this?** YES (give details) / No / N/A

If yes, please state what type or give details below:-

Handout material (not relevant if just poster handout or summary) / Links for download / Example materials shown

_________________________________________________________________________

_________________________________________________________________________

_________________________________________________________________________

**If yes, please state on a scale of 1–5 how useful this is, 1 being limited and not of great benefit, 5 supporting widespread replication**.

_________________________________

## References

[1] Todres M, Stephenson A, Jones R (2007). Medical education research remains the poor relation. BMJ.

[2] Dornan T, Peile JS, Spencer J (2008). On ‘evidence’. Med Educ.

[3] Eva KW (2009). Broadening the debate about quality in medical education research. Med Educ.

[4] Gordon M, Darbyshire D, Baker P Separating the wheat from the chaff … the role of systematic review in medical education. Med Educ.

[5] http://www.amee.org/documents/AMEE%202012%20ABSTRACT%20BOOK.pdf.

